# Mangiferin as a Novel In Vitro Polyphenolic Inhibitor of Amyloid Aggregation

**DOI:** 10.1021/acsomega.5c06703

**Published:** 2025-10-28

**Authors:** Daniele Florio, Enrico Gallo, Anella Saviano, Anna Schettino, Noemi Marigliano, Ilaria Leone, Francesco Maione, Daniela Marasco

**Affiliations:** † 591458 IRCSS SYNLAB SDN, Via G. Ferraris 144, Naples 80146, Italy; ‡ ImmunoPharmaLab, Department of Pharmacy, School of Medicine and Surgery, 165474 University of Naples Federico II, Via Domenico Montesano 49, Naples 80131, Italy; § Nutraceuticals and Functional Foods Task Force, University of Naples Federico II, Via Domenico Montesano 49, Naples 80131, Italy; ∥ Department of Pharmacy, School of Medicine and Surgery, University of Naples Federico II, Via Domenico Montesano 49, Naples 80131, Italy

## Abstract

Amyloid aggregation is a pathological hallmark of several neurodegenerative disorders, including Alzheimer’s disease. Polyphenolic compounds are emerging as promising candidates for therapeutic intervention due to their capacity to interfere with multiple stages of amyloidogenesis. In this study, we investigated, *in vitro*, the antiamyloidogenic potential of mangiferin (**MGF**), a xanthonoid polyphenol with established pharmacological activity but previously unexplored in the context of amyloid modulation. Using a combination of biophysical, spectroscopic, and microscopic techniques, we assessed the effects of **MGF** on the aggregation behavior of two distinct amyloidogenic peptides: Aβ_1–42_ and Cterm_mutA. Thioflavin T (ThT) assays revealed that **MGF** significantly inhibited aggregation in a concentration-dependent manner, with maximal inhibition at a 1:5 peptide:**MGF** ratio. Nanoparticle tracking analysis (NTA) and microscopy studies demonstrated peptide-specific differences in the mechanism of action of **MGF**: **MGF** promoted the formation of larger, nonfibrillar oligomers in Aβ_1–42_, while it reduced oligomer size in Cterm_mutA. This effect was most likely attributable to the disruption of π–π interactions. Importantly, **MGF** exhibited no cytotoxicity in SH-SY5Y cells and significantly attenuated the amyloid-induced toxicity of both peptides. These findings highlight **MGF** as a promising, multitargeted modulator of amyloid aggregation with potential applications in neuroprotection and the development of novel antiamyloid therapies

## Graphical abstract

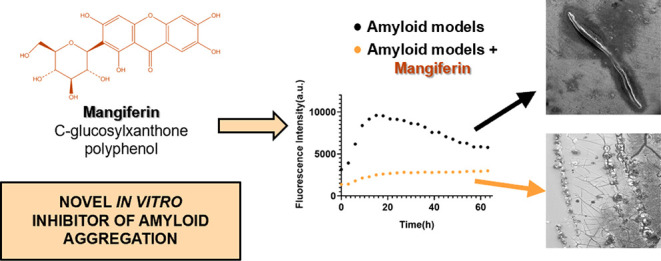

## Introduction

Protein aggregation is a hallmark of several misfolding disorders, including Alzheimer’s (AD),^1^ Parkinson’s (PD),^2^ and Huntington’s diseases (HD).^3^ Consequently, developing effective therapeutic strategies to prevent amyloid aggregation is crucial. Over time, numerous inhibitors of amyloid aggregation as antibodies,^4^ peptides,^5^ organic molecules,^6^ metal complexes,^7^ and nanoparticles^8^ have been designed to selectively delay and/or suppress the formation of toxic amyloid species at different stages of aggregation, including intermediate oligomeric forms and mature fibrils.^9^ In this context, natural bioactive compounds derived from food sources have attracted considerable attention in recent years,^10^ particularly polyphenols, due to their distinctive physicochemical properties.^11–13^ Polyphenols are molecules containing one or more phenolic aromatic rings and exhibit a broad spectrum of bioactive properties, including antioxidant, antimicrobial, anticancer, antidiabetic, anti-inflammatory and neuroprotective activities.^14–16^ In several cases, they demonstrated to be able to cross the blood–brain barrier (BBB),^17^ making them attractive as therapeutic agents.^17–19^ Among their bioactive properties, polyphenols are particularly recognized for having a marked capacity for redox homeostasis modulation.^14^ They act as free radical scavengers,^20^ chelators of transition metal ions and binders of cell membranes or other biomolecules.^21^ Furthermore, they modulate enzymatic metabolism by inducing, activating, inhibiting, or protecting oxidase enzymes such as lipoxygenase (LO), cyclooxygenase (COX), myeloperoxidase (MPO), nicotinamide adenine dinucleotide phosphate (NADPH) oxidase (NOX) and xanthine oxidase (XO).^22^ Polyphenols protect cellular components by stabilizing radical intermediates through resonance delocalization, thereby preventing oxidative damage and lipid peroxidation.^23,24^ *In vitro* studies showed the potential of various polyphenolic compounds to act as modulators of amyloid aggregation for their ability to directly interact with amino acid and peptide backbone:^25–27^ hydroxyl groups and aromatic rings of polyphenols can interfere with protein self-association by establishing both H-bond and aromatic interactions.^28^ For instance, brazilin-7–2-butenoate (B-7–2-B), a derivative of the natural compound brazilin, exhibited strong inhibitory effects on Aβ_1–42_ and Aβ_1–40_ aggregation^29^ preventing oligomer formation in a concentration-dependent manner, reducing oxidative stress and cytotoxicity induced by Aβ aggregates in neuronal cells, as well as alleviating behavioral and sensory deficits caused by Aβ aggregation in AD *Caenorhabditis elegans* models.^29^ Similarly, bisdemethoxycurcumin (BDMC), a curcuminoid, decreased the development of β-sheet structures and efficiently suppressed aggregation linked to the ALS-related SOD1 mutant (L38R).^30^ In addition, polyphenols such as forsitoside B (FTS·B) and echinacoside (ECH) have recently demonstrated an attractive antiaggregating ability:^31^ both demonstrated to prevent α-synuclein aggregation by modulating liquid–liquid and liquid–solid phase separation pathways and to reduce its toxicity in neuronal cells.^32^

Mangiferin (2-β-d-glucopyranosyl-1,3,6,7-tetrahydroxy-9H-xanthen-9-one, **MGF**) (Figure 1A) is a naturally occurring C-glucosylxanthone polyphenol, primarily found in *Mangifera indica*. Structurally, it consists of a xanthone core substituted with four hydroxyl groups and a β-d-glucopyranosyl moiety at the C-2 position, making it a C-glycoside rather than an O-glycoside. This feature confers it greater stability against enzymatic hydrolysis.^33^ **MGF** exhibits a broad spectrum of pharmacological properties^34–36^ that are largely attributed to its ability to modulate key intracellular signaling pathways, such as mitogen-activated protein kinase (MAPK), nuclear factor erythroid 2-related factor 2 (Nrf2), nuclear factor-kappa B (NF-κB), adenosine monophosphate-activated protein kinase (AMPK) and the mammalian target of rapamycin (mTOR).^37,38^

**Figure 1:**
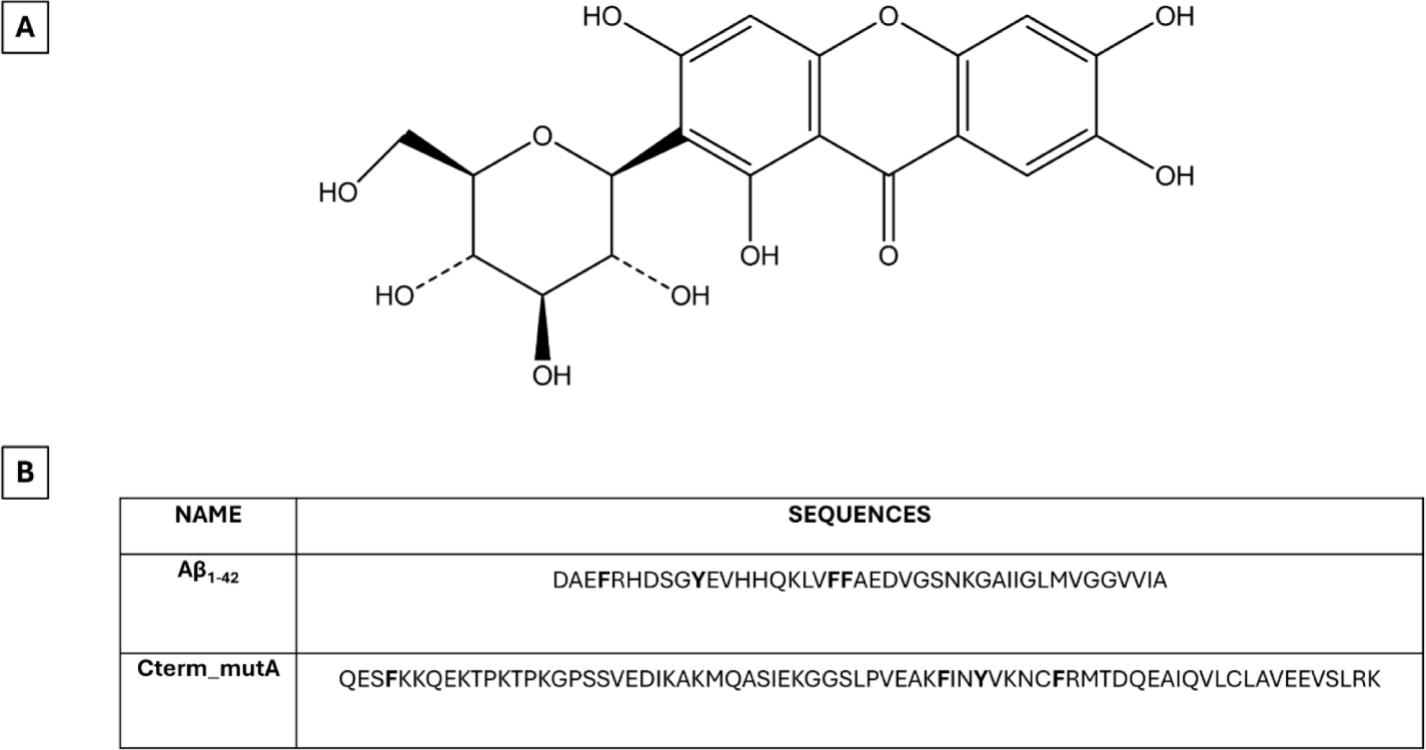
(**A**) Chemical structure of **MGF** compound and (**B**) primary sequences of Aβ_1–42_ and Cterm_mutA polypeptides analyzed in this work. Aromatic amino acids are highlighted in bold.

Several studies have demonstrated that **MGF** exerts potent neuroprotective effects, primarily through the modulation of key signaling pathways involved in cellular survival,^39^ oxidative stress response^40^ and inflammation.^41^ Indeed, **MGF** regulates the phosphoinositide 3-kinase/protein kinase B (PI3K/Akt), Nrf2/heme oxygenase-1 (HO-1) and extracellular signal-regulated kinase 1/2 (ERK1/2) pathways, which play crucial roles in neuronal survival and the cellular antioxidant defense system.^38^ **MGF** significantly inhibits the activation of NF-κB and prevents the degradation of its inhibitory protein, IκB. By doing so, it regulates the transcription of a wide array of genes, including those encoding pro-inflammatory cytokines and mediators of neuroinflammation.^42^ Moreover, **MGF** effectively suppresses the expression of interleukin (IL)-6 and IL-1β, two major pro-inflammatory cytokines known to enhance NF-κB signaling and contribute to neurodegenerative processes.^43^ It also attenuates the activation of the nucleotide-binding oligomerization domain (NOD)-like receptor family pyrin domain containing 3 (NLRP3) inflammasome, a key component in the innate immune response implicated in chronic neuroinflammation in AD and PD.^44^ Importantly, **MGF** was demonstrated to cross the BBB by exerting a direct neuroprotective action within the central nervous system (CNS). This includes protection against dopaminergic neuronal cell death, a hallmark feature of PD, highlighting its potential as a therapeutic agent in neurodegenerative disorder treatment.^45^

With the aim of investigating the ability of **MGF** to directly modulate amyloid aggregation, two amyloid models were selected: (i) Aβ_1–42_ and (ii) Cterm_mutA. The Aβ_1–42_ polypeptide (Figure 1B) is a cleavage product of the amyloid precursor protein (APP), generated by β- and γ-secretases cleavage.^46,47^ It is one of the main components of amyloid deposits found in the brains of patients with AD.^48,49^ Conversely, the Cterm_mutA is a “not neurodegenerative” polypeptide (Figure 1B) since it is the C-terminal domain (CTD) of nucleophosmin 1 (NPM1) protein in its type A mutation. This mutation is the most common in Acute Myeloid Leukemia (AML) patients.^50,51^ NPM1 is not a “neurodegenerative protein” as traditionally defined, but many studies^52–59^ demonstrated that AML mutations determine a great propensity to amyloid aggregation. Since Aβ_1–42_ and Cterm_mutA have demonstrated differences in both the kinetics and morphology of the fibers^60–62^ they were employed as amyloid models in this study. Herein, a wide range of spectroscopic, biophysical, and microscopic techniques, as well as cellular assays, were employed to demonstrate the potential ability of **MGF** to act as an inhibitor of amyloid aggregation.

## Results and Discussion

### Kinetic Effects of MGF on Aβ_1–42_ and Cterm_mutA Aggregation: ThT Assay

The effects of **MGF** on the self-aggregation process of Aβ_1–42_ and Cterm_mutA polypeptides were investigated by the Thioflavin T (ThT) assay.^63,64^ The time course profiles of ThT fluorescence for Aβ_1–42_ and Cterm_mutA, alone and in the presence of **MGF**, at the indicated molar ratios, are shown in Figure 2. The nonzero ThT fluorescence values observed at *t* = 0 for both polypeptides may indicate the presence of a partially preaggregated fraction within the initial samples. The corresponding *t*_1/2_ values (the time at which ThT fluorescence reaches half of its maximum value), maxima of ThT intensity and percentages of inhibition are reported in Table 1. The results indicated that the presence of **MGF** significantly alters the kinetic profiles of the two polypeptides, with distinct variations depending on the molar ratio peptide: **MGF**. Aβ_1–42_ alone exhibited a typical sigmoidal time course of fluorescence, with a *t*_1/2_ of 2.5 h and a maximum intensity of 414 au. The addition of **MGF** induced a concentration-dependent reduction of signals, with the most significant effect observed at a 1:5 molar ratio, which provided a maximum fluorescence value of 204 au with 51% of inhibition. Similarly, Cterm_mutA showed clear self-aggregation kinetics with a maximum intensity of 257 au with a *t*_1/2_ of 10 h. The addition of **MGF** caused a decrease in fluorescence intensity at all three analyzed ratios. The reduced ThT fluorescence observed at *t* = 0 for 1:5 ratio suggests that the interaction between MGF and the amyloid species (already partially aggregated) may alter ThT binding properties even prior to the progression of aggregation and that MGF exerts an “almost immediate” inhibitory effect. Additionally, a reduction in *t*_1/2_ was detected for 1:1 and 1:2 ratios (Table 1). At the 1:5 ratio, an inhibition of 58% was observed, while control experiments confirmed negligible interference of **MGF** with ThT fluorescence.

**Figure 2:**
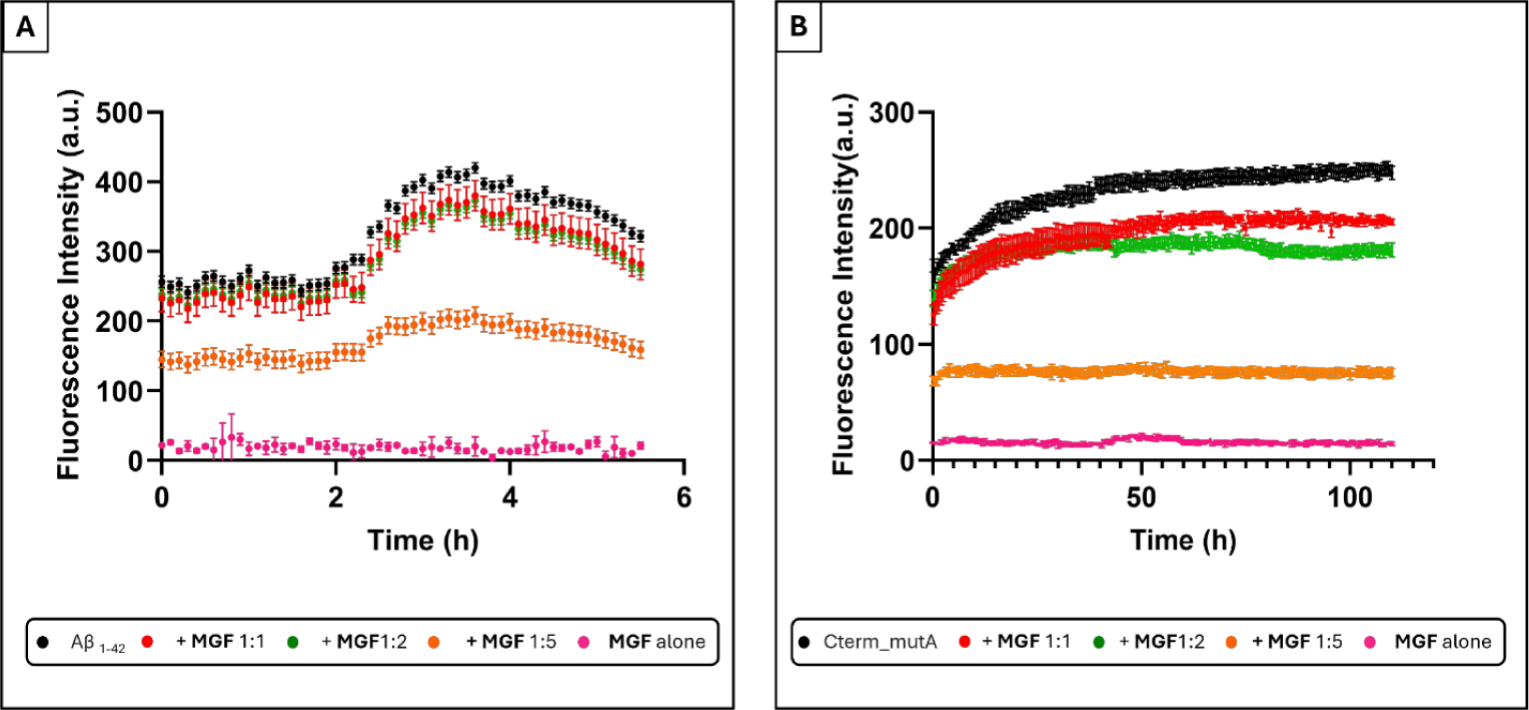
Overlay of time courses of ThT fluorescence emission intensity of: **A**) Aβ _1–42_ in the absence and in the presence of **MGF** at 1:1, 1:2, and 1:5 peptide-to-compound molar ratio; (**B**) Cterm_mutA in the absence and presence of **MGF** at 1:1, 1:2 and 1:5 peptide-to-compound molar ratios. **MGF** alone was also assessed at 250 μM and 400 μM, corresponding to the highest concentrations used with Aβ_1–42_ and Cterm_mutA, respectively. Data points represent mean values and error bars indicate standard deviations from three independent experiments.

**Table 1:** Experimental Values of *t*_1/2_, Maximum Fluorescence Intensity, % of Inhibition, Sizes of Aggregates from SEM and NTA Analyses of Aβ_1–42_ and Cterm_mutA Polypeptides in the Absence and in the Presence of MGF^a^

				SEM Analysis		NTA Analysis Average Diameters (nm) (Peaks)					
Samples	t_1/2_ (h)	Maximum Intensity (a.u.)	% Inhibition	Diameter (μm)	Length (μm)	1^st^	2^nd^	3^rd^	4^th^	5^th^	6^th^
Aβ_1–42_											
	2.5	414	/	23.2 ± 0.8	1231 ± 5	70	107	165			
: **MGF** 1:1	2.6	373	10	n.e.	n.e.						
: **MGF** 1:2	2.7	361	13	n.e.	n.e.						
: **MGF** 1:5	n.e.	204	51	no fiber		47	72	102	150	237	328
Cterm_mutA											
	14	257	/	13 ± 2	1404 ± 3	75	165	260			
: **MGF** 1:1	10	205	20								
: **MGF** 1:2	6.5	182	29								
: **MGF** 1:5	n.e.	109	58	no fiber		34	96	122	165		

a n.e. not evaluated.

### Effects of MGF on the Oligomeric States of the Aβ_1–42_ and the Cterm_mutA: Nanoparticle Tracking Analysis (NTA)

The effects of **MGF** on the aggregation of the polypeptides were analyzed using NTA analysis.^65^ This method relies on the detection of light scattering from individual particles and tracking of their trajectories over a brief period (from 30 s to 5 min). Compared to conventional Dynamic Light Scattering (DLS), NTA offers distinct advantages: it enables the quantification of particle number concentration, discriminates among small and weak scatterers in the presence of larger scatterers and provides a size distribution by analyzing the motion of individual particles. NTA experiments were carried out at a 1:5 peptide:**MGF** molar ratio, after 2 h of stirring, the size distribution of oligomers is reported in Table 1.

The NTA analysis of Aβ_1–42_ alone (Figure 3A) showed two peaks centered at 70 and 110 nm in accordance with previous analyses already reported;^66^ the presence of **MGF** determined: (i) a marked reduction in the total concentration (particles mL^–1^) of aggregates; (ii) a shift of the predominant peak toward larger diameters with respect to Aβ_1–42_ alone; (iii) a more heterogeneous population of oligomers. This heterogeneity is reflected in the appearance of multiple distinct peaks of different sizes (Table 1). Similar inhibitory effects on Aβ_1–42_ aggregation have also been reported for other natural polyphenols, such as epigallocatechin gallate (EGCG), which significantly reduced aggregate concentration and altered the distribution of oligomeric species.^66^

**Figure 3:**
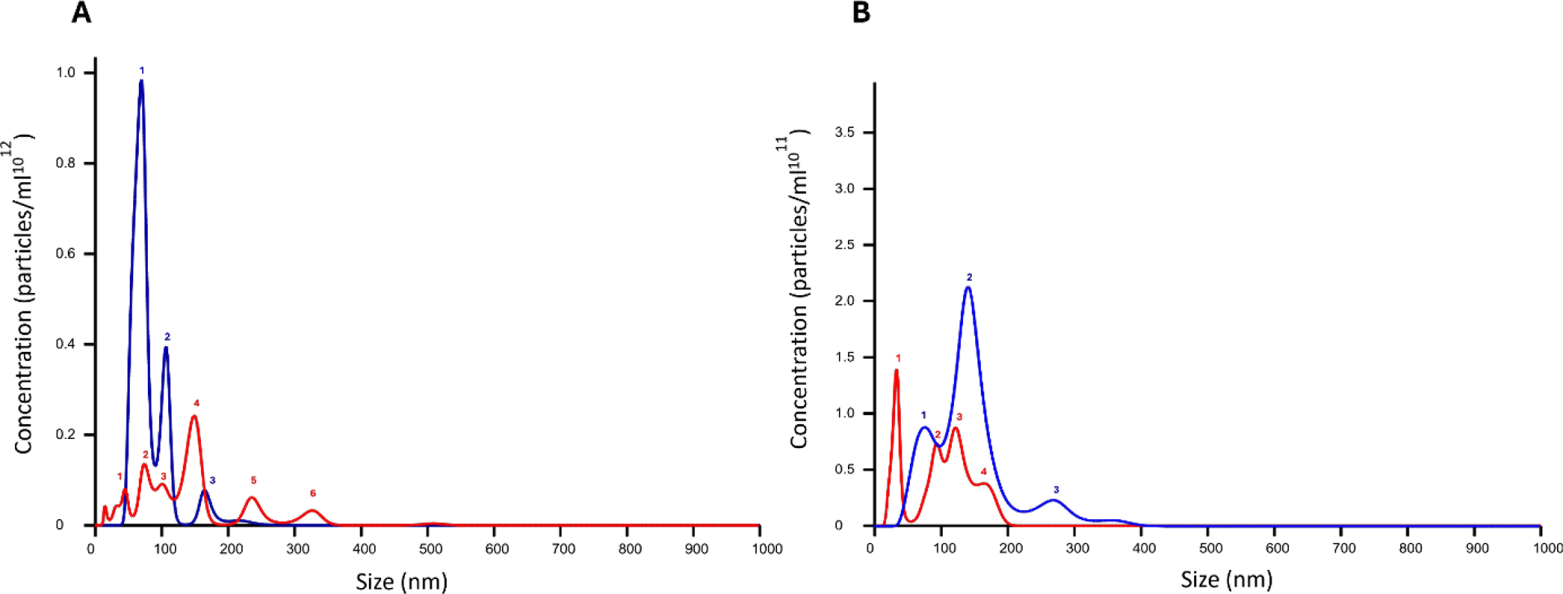
Size distribution of particles using NTA of: (**A**) Aβ_1–42_ and (**B**) Cterm_mutA in the absence (blue) and in the presence of **MGF** (red). Numbers refer to the peaks whose sizes are given in Table 1.

Cterm_mutA was analyzed, for the first time, by means of NTA and exhibited three main peaks with diameters of 76, 165 and 260 nm after 2 h of aggregation, which are in reasonable agreement with those detected with DLS after 17 h of aggregation.^62^ Also, in this case, the presence of **MGF** (Figure 3B) determined a reduction in the aggregate concentrations but caused a shift toward lower diameters with respect to the polypeptide alone. These results indicate that, in solution, **MGF** alters the aggregation of amyloid models to two different extents: for Aβ_1–42_ stabilized larger aggregates, while for Cterm_mutA smaller species.

### MGF Suppresses Fibril Formation: Microscopy Experiments

To get insights into the effects of **MGF** on the morphology of the fibers derived from Aβ_1–42_ and Cterm_mutA, Scanning Electron Microscopy (SEM) images were recorded at two different times based on the *t*_1/2_ values of polypeptides (Table 1): 24 h for Aβ_1–42_ and 48 h of aggregation for Cterm_mutA, at 1:5 peptide:**MGF** molar ratio. As reported in Figure 4, well-defined fibers were observed for both Aβ_1–42_ (Figure 4A,A’) and Cterm_mutA (Figure 4B,B’) alone as already reported.^60,61^ Specifically, Aβ_1–42_ peptide provided a fiber with an average length of ∼1 mm and a diameter of ∼20 μm (Figure 4A,A’ and Table 1) while in the case of Cterm_mutA, the fibers had an average length of ∼1.4 mm and a diameter of ∼13 μm.

**Figure 4:**
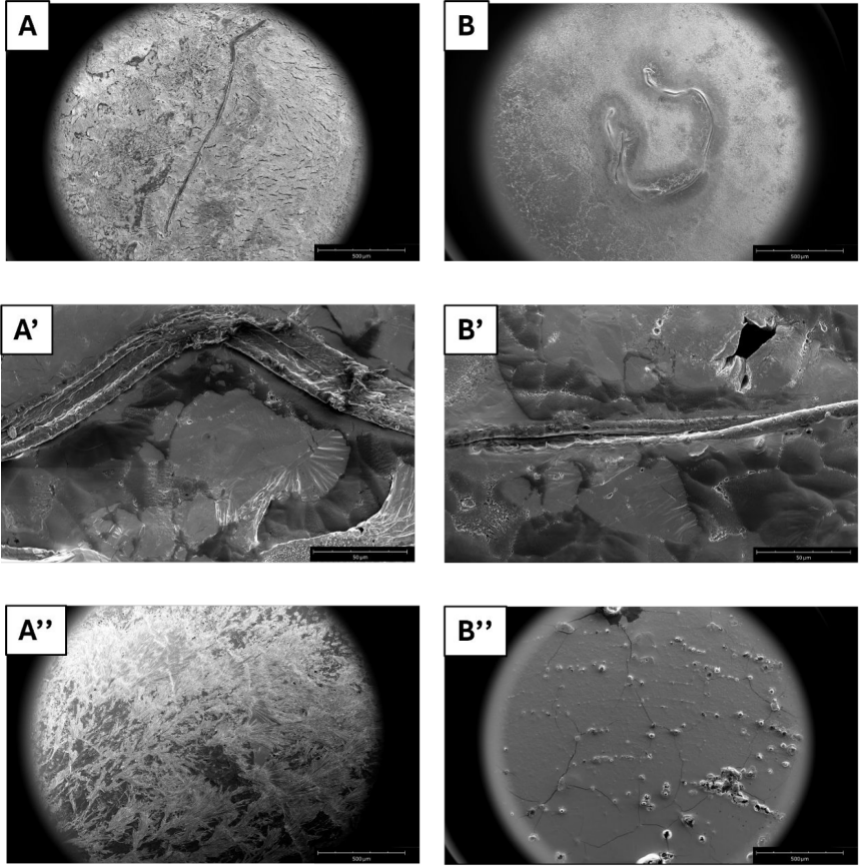
SEM micrographs of: (**A-A’**) Aβ_1–42_ alone and (**A’’**) Aβ_1–42_ + **MGF** after 24 h of aggregation; (**B–B’**) Cterm_mutA alone and (**B’’**) Cterm_mutA + **MGF** after 48 h. Surface overviews at 500 μm (**A**, **A’’**, **B**, **B’’**) and 50 μm (**A’’** and **B’’**).

The presence of **MGF** resulted in the complete suppression of fibrillization, with no aggregates observed (Figure 4A’’,B’’) like **MGF** alone (Figure S1A,B). To further corroborate the inhibitory effects of **MGF** on amyloid fibers, label-free microscopy was employed^67^ and the images were captured both in the blue-emission region (Figure S2A,B)^68^ and in the bright field (Figure S3A-C) after 24 h of aggregation for Aβ_1–42_ and 48 h for Cterm_mutA, in the presence or absence of **MGF**. Aggregates derived from both polypeptides displayed blue light emission, indicating the presence of amyloid fibers (Figure S2A,B). In contrast, the presence of **MGF** completely suppressed this emission (Figure S2A,B). Bright-field images (Figure S3A,C) further confirmed these observations.

### Cellular Effects of MGF on the Amyloid Cytotoxicity Driven by Aβ_1–42_ and Cterm_mutA

The cytotoxicity of **MGF** was already investigated in murine macrophage J774A.1 cell line.^41^ Since SH-SY5Y is a well-established human neuroblastomal cell line sensitive to amyloid toxicity, to preliminary evaluate the effects of **MGF**, a 3-(4,5-dimethylthiazol-2-yl)-2,5-diphenyltetrazolium bromide (MTT) cell viability assay was performed on SH-SY5Y cells treated with different concentrations of **MGF** (up to 400 μM at 0 and 24 h). **MGF** exhibited no significant cytotoxicity under the tested conditions (Figure S4), indicating that **MGF** is well-tolerated by SH-SY5Y cells within the concentration range evaluated, thereby supporting its suitability for further investigation as a neuroprotective agent. The neuroprotective potential of **MGF** against amyloid-induced cytotoxicity of Aβ_1–42_ and Cterm_MutA polypeptides at two different time points (0 and 24 h) and two different molar ratios (1:1 and 1:5 peptides:**MGF**) was assessed. As reported in Figure 5, both polypeptides alone and untreated showed cytotoxic effects: in the case of Aβ_1–42_, cell viability was significantly reduced compared to untreated controls, with the effect being more pronounced after 24 h of preaggregation with a cell viability <75%, consistent with already reported studies^61,69^ where prolonged aggregation increased the toxicity of the peptide (Figure 5A,B).

**Figure 5:**
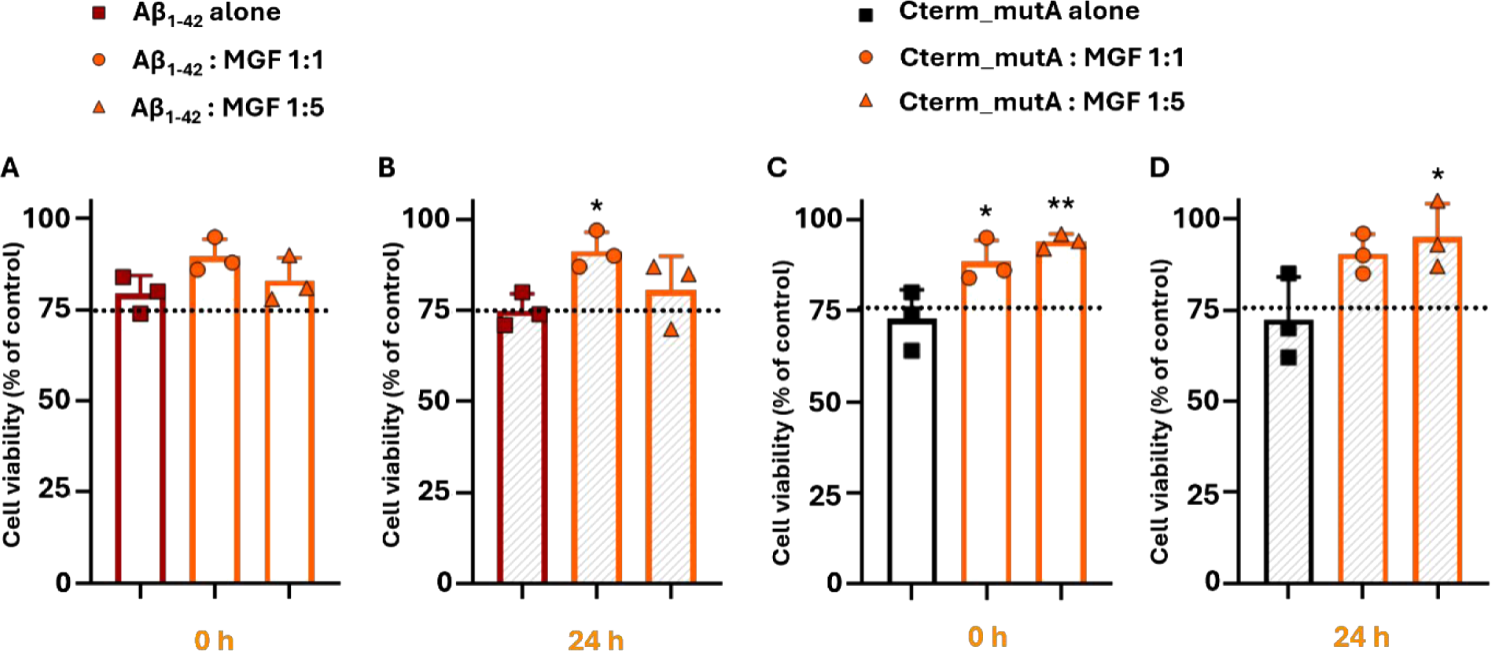
Effect of **MGF** in cell viability of SH-SY5Y human neuroblastoma cells treated with Aβ_1–42_ alone (50 μM) and Aβ_1–42_:**MGF** at 1:1 and 1:5 peptide-to-compound molar ratios after stirring for 0 and 24 h was evaluated (**A** and **B**). Similarly, we assessed the protective effect of **MGF** in SH-SY5Y cells treated with Cterm_mutA peptide alone (80 μM) and Cterm_mutA:**MGF** at 1:1 and 1:5 peptide-to-compound molar ratios under the same conditions (**C** and **D**). The dotted lines indicate the threshold for 75% cell viability. Cell viability (% of control) is presented as the mean ± SD of three independent experiments. Statistical analysis was performed using one-way ANOVA with Bonferroni’s multiple comparisons test. *p ≤ 0.05, **p ≤ 0.01 vs Aβ_1–42_ alone or Cterm_mutA alone at the corresponding time points.

Similarly, Cterm_mutA induced cytotoxicity at 0 and 24 h, with cell viability falling <72% in both cases (Figure 5C-D). The incubation with **MGF** slightly mitigated the cytotoxic effects of both polypeptides. Specifically, for Aβ_1–42_, the condition 1:1 peptide:**MGF** molar ratio, after 24 h, led to a recovery of cell viability (mean cell viability 91%, *p* ≤ 0.05), indicating that **MGF** can counteract the toxicity of Aβ species (Figure 5A-B). The lack of a clear dose–response effect in the case of Aβ_1–42_, suggested the occurrence of other factors that can affect cell viability. Furthermore, for Cterm_mutA, **MGF** demonstrated an more pronounced effect since an increase in cell viability was observed at 1:1 molar ratio peptide:**MGF** after 0 h (mean cell viability 88%, *p* ≤ 0.05), with a further enhancement at the 1:5 molar ratio (mean cell viability 94%, *p* ≤ 0.01) (Figure 5C). Notably, this effect remained significant at the 1:5 ratio even at 24 h (mean cell viability 95%, *p* ≤ 0.05) (Figure 5D).

## Experimental Section

### Reagents

Dimethyl sulfoxide (DMSO), fetal bovine serum (FBS), (4-(2-hydroxyethyl)-1-piperazineethanesulfonic acid (HEPES) buffer solution and **MGF** were purchased from Sigma-Aldrich Co. (now under Merck, Darmstadt, Germany). Dulbecco’s modified Eagle’s medium (DMEM) was obtained from Corning. 3-(4,5-dimethyl-2-thiazolyl)­2,5-diphenyl-2H-tetrazolium bromide (MTT) was purchased from BioBasic. Unless otherwise stated, all other reagents were obtained from BioCell (Milan, Italy).

### Preparation of MGF for *In Vitro* Assays

**MGF** (Product No. M3547, CAS No. 4773–96–0, Merck, Darmstadt, Germany) was dissolved in DMSO to prepare concentrated stock solutions.^41,70^ To minimize the final concentration of DMSO in cell culture assays, mother stocks were prepared at concentrations four times higher than the desired working concentrations. Specifically, for experiments involving Aβ_1–42_, stock solutions of 200 and 1000 μM were used to achieve final concentrations of 50 and 250 μM, respectively. For Cterm_mutA experiments, stock solutions of 320 μM and 1600 μM were prepared to obtain final concentrations of 80 μM and 400 μM. All stock solutions were freshly prepared and diluted in culture medium immediately prior to use. The final DMSO concentration in each well was carefully controlled to remain below levels known to induce cytotoxicity or exert pharmacological activity *per se.* Vehicle controls were included in all experiments and prepared under identical conditions to account for any solvent-related effects.

### Polypeptides

The Aβ_1–42_ and Cterm_mutA polypeptides (sequences are reported in Figure 1B) were purchased from NovoPro Bioscience Inc. (Shanghai, China). Both peptides were treated with 1,1,1,3,3,3-hexafluoro-2-propanol (HFIP) to guarantee a monomeric state, lyophilized and stored at −20 °C until use.

### Fluorescence Assays

ThT emission assays of Aβ_1–42_ alone (50 μM) and in the presence of **MGF** at 1:5 peptide:compound molar ratio in 50 mM NaCl, 20 mM phosphate buffer (pH 7.4)/DMSO 2% (v/v), using a ThT final concentration of 5 μM, were carried out in black plates (96-well) under stirring on a fluorescence reader Envision 2105 (PerkinElmer). Measurements were collected every 7 min (λex= 440 nm and λem= 483 nm). Assays were performed in duplicate at 25 °C. For Cterm_mutA alone (80 μM) and in the presence of **MGF** at 1:5 peptide:compound molar ratio, the experiments were performed in 50 mM phosphate buffer (pH 7.4)/DMSO 2% and 50 μM of ThT and were analyzed using a CLARIOstar fluorescence microplate reader (BMG Labtech) at 25 °C in black, clear-bottomed 96-well half-area polystyrene plates with nonbonding surface (Corning #3881) covered with aluminum thermowell sealing tape (Corning #6570). The experiments were performed in 100 μL aliquots in triplicate.

### NTA Measurements

The NTA measurements were carried out using a nanosight NS300 instrument (Alfatest, Italy). Samples of Aβ_1–42_ and Cterm_mutA at a concentration of 100 μM, in the absence and in the presence of **MGF** (1:5 peptide:compound molar ratio), after 2 h of aggregation, were 1000-fold diluted in Milli-Q water to a final volume of 1 mL and were injected into the sample chamber using a syringe. The dilution was done in accordance with the ideal particle-per-frame value (20–100 particles/frame). The following settings were chosen according to the manufacturer’s software manual (NanoSight NS300 User Manual, MAN0541–01-EN-00, 2017).^71^

### SEM Analysis

The two polypeptides, Aβ_1–42_ (50 μM) and Cterm_mutA (80 μM), alone and in the presence of **MGF** compound (1:5 peptide:compound molar ratio), were morphologically analyzed after 24 and 48 h of aggregation, respectively, using field-emission SEM (Phenom_XL, Alfatest, Milan, Italy). After this time, 50 μL of solution was drop-cast on an aluminum stub and dried under vacuum to prepare the samples. For 75 s, a thin layer of gold was sputtered at a current of 25 mA. Following the introduction of the sputter-coated samples into the specimen chamber, micrographs were taken using a secondary electron detector (SED) at an accelerating voltage of 10 kV. **MGF** compound alone (500 μM) was analyzed as a control.

### Fluorescence Microscopy

Aβ_1–42_ and Cterm_mutA samples employed for the SEM experiments were drop-cast on clean coverslip glass, dried and imaged with fluorescence microscopy. Fluorescence images were captured with an automated upright microscope system (Leica DM5500 B) coupled with Leica Cytovision software.

### Cell Culture

SH-SY5Y human neuroblastoma cell lines (CRL-2266, ATCC, Manassas, VA, USA) were cultured in 100 × 20 mm dishes (1 ×10^6^ cells/dish) in DMEM (Corning; Product No. 10–013-CV) supplemented with 10% FBS (Sial; Product No. YourSIAL-FBS-SA), 100 U mL^–1^ penicillin, 100 μg mL^–1^ streptomycin (Corning; Product No. 30–002-CI) and 25 mM HEPES (Sigma-Aldrich; Product No. H0887). Cells were maintained in a humidified atmosphere of 5% CO_2_ at 37 °C and passaged upon reaching 80% confluence.^72,73^

### MTT Assay

The ability of **MGF** to reduce the neurotoxicity of Aβ_1–42_ and Cterm_mutA polypeptides was evaluated in a human neuroblastoma cell line by using the MTT assay. As a preliminary step, the assay was first performed to assess whether **MGF** exerted any cytotoxic effects on this specific cell line. To this aim, SH-SY5Y cells were seeded at a density of 2.5 × 10^4^ cells per well in 96-well plates, allowed to adhere overnight, and subsequently treated with **MGF** at concentrations of 50, 80, 250 and 400 μM (after 0, 24 and 48 h of stirring), with cell viability assessed at 24 h time-point. Subsequently, cells were treated with Aβ_1–42_ peptide (50 μM), either alone or in combination with **MGF** at 1:1 and 1:5 peptide:compound molar ratios (after 0 and 24 h of stirring). Likewise, the Cterm_mutA peptide (80 μM) was tested under the same conditions. Control cells were incubated with DMSO diluted in the cell culture medium at the same % used for treatments. At the selected time point (24 h), 10 μL of MTT (BioBasic; Product No. T0793) solution (5 mg mL^–1^ in phosphate-buffered saline, PBS; pH 7.4) was added to each well and the plates were incubated for 3 h at 37 °C in the dark. Then, the medium was removed and the resulting formazan crystals were dissolved in 150 μL DMSO for 15 min. The spectrophotometric absorbance was measured using a microtiter enzyme-linked immunosorbent assay reader (Multiskan GO Microplate Spectrophotometer; Thermo Scientific) at 540 nm. The percentage of cell viability was determined by the following formula: OD of treated cells/OD of control × 100.^74^ Each experiment was performed in biological triplicate.

### Statistical Analysis

All statistical analyses were performed in accordance with established guidelines for experimental design, data analysis and transparent reporting. Data from three independent experiments (*n* = 3) are reported as the mean ± standard deviation (SD). For ThT fluorescence assays, error bars indicate the SD of three independent replicates. For cytotoxicity assays, statistical significance between groups was assessed by one-way ANOVA followed by Bonferroni’s post hoc test for multiple comparisons, with *p* ≤ 0.05 considered significant. Analyses were performed using GraphPad Prism 8.0 (GraphPad Software, San Diego, CA, USA).

## Conclusions

In this study, we aimed to evaluate the antiamyloidogenic activity of **MGF** using a multitiered approach that combined spectroscopic and microscopic methodologies with cellular assays. We investigated its effects on the aggregation behavior of two structurally distinct amyloidogenic peptides: Aβ_1–42_, a prototypical peptide implicated in AD and Cterm_mutA, a synthetic model peptide characterized by aggregation driven predominantly through π–π interactions.

ThT fluorescence assays revealed that **MGF** exerts an inhibitory effect on amyloid aggregation for both peptides, with maximal inhibition observed at a 1:5 peptide:**MGF** molar ratio. This ratio was subsequently employed in NTA and electron microscopy studies. Despite the distinct aggregation mechanisms of Aβ_1–42_ and Cterm_mutA-driven predominantly by hydrophobic/electrostatic interactions and aromatic stacking, respectively, **MGF** effectively inhibited aggregation in both cases. The data suggest that **MGF** could disrupt peptide self-association through multiple interaction modalities: its aromatic scaffold is likely critical for disrupting π-stacking in Cterm_mutA, while its polyhydroxylated structure facilitates hydrogen bonding and electrostatic interference in Aβ_1–42_ fibrillogenesis.^75,76^ This hypothesis, however, requires further, more detailed structural studies. These differences were further supported by NTA and microscopy data, which showed a stabilization of larger, nonfibrillar oligomeric species in the case of Aβ_1–42_, consistent with fibrillation arrest and a marked reduction in oligomer size for Cterm_mutA, consistent with disruption of aromatic interactions.

Importantly, **MGF** displayed no intrinsic cytotoxicity in SH-SY5Y neuroblastoma cells. Moreover, cotreatment with **MGF** slightly ameliorated the cytotoxic effects induced by both Aβ_1–42_ and Cterm_mutA aggregates, underscoring its neuroprotective potential.

In conclusion, our findings suggest **MGF** as a starting-point molecule to develop modulators of amyloid aggregation, with potential for applications in neuroprotection, neurodiagnostics and the development of novel antiamyloid therapies. To fully translate these findings, comprehensive preclinical and *in vivo* studies are now critically needed to elucidate the complete therapeutic potential and mechanisms of action.

## Supplementary Material



## Notes

SEM micrographs after 48 h of aggregation of **MGF** (**Figure S1**) Fluorescence microscopy of Aβ_1-42_ and Cterm_mutA with and without **MGF** Bright field microscopy of Aβ_1-42_ and Cterm_mutA with and without **MGF** and **MGF** alone (**Figure S3**) *In vitro* cytotoxicity (MTT assay) of **MGF** on SH-SY5Y cells (**Figure S4**) (PDF)

## Abbreviations

2-β-d-glucopyranosyl-1,3,6,7-tetrahydroxy-9H-xanthen-9-one: mangiferin
AD: Alzheimer’s disease
AML: acute myeloid leukemia
B-7–2-B: brazilin-7–2-butenoate
BDMC: bisdemethoxycurcumin
BBB: blood–brain barrier
CNS: central nervous system
COX: cyclooxygenase
CTD: C-terminal domain
DLS: dynamic light scattering
DMEM: Dulbecco’s modified Eagle’s medium
DMSO: dimethyl sulfoxide
ECH: echinacoside
EGCG: epigallocatechin gallate
ERK1/2: extracellular signal-regulated kinase 1/2
FBS: fetal bovine serum
FTS·B: forsitoside B
HEPES: 4-(2-hydroxyethyl)-1-piperazineethanesulfonic acid
HFIP: 1,1,3,3,3-hexafluoro-2-propanol
HD: Huntington’s disease
LO: lipoxygenase
MAPK: mitogen-activated protein kinase
MPO: myeloperoxidase
MTT: 3-(45-dimethyl-2-thiazolyl)­2,5-diphenyl-2H-tetrazolium bromide
mTOR: mammalian target of rapamycin
NADPH oxidase (NOX): nicotinamide adenine dinucleotide phosphate oxidase
NFKB: nuclear factor-kappa B
NLRP3: nucleotide-binding oligomerization domain-like receptor family pyrin domain containing 3
NPM1: nucleophosmin 1
Nrf2: nuclear factor erythroid 2-related factor 2
NTA: nanoparticle tracking analysis
NOD: nucleotide-binding oligomerization domain
PD: Parkinson’s disease
PI3K/Akt: phosphoinositide 3-kinase/protein kinase B
SED: secondary electron detector
SEM: scanning electron microscopy
ThT: Thioflavin T
XO: xanthine oxidase

## References

[1] Murphy M. P., LeVine H. (2010). Alzheimer’s disease and the
amyloid-β peptide. J. Alzheimers Dis..

[2] Calo L., Wegrzynowicz M., Santivañez-Perez J., Grazia Spillantini M. (2016). Synaptic failure
and α-synuclein. Mov. Disord..

[3] Arrasate M., Finkbeiner S. (2012). Protein aggregates
in Huntington’s disease. Exp. Neurol..

[4] Haddad H. W., Malone G. W., Comardelle N. J., Degueure A. E., Poliwoda S., Kaye R. J., Murnane K. S., Kaye A. M., Kaye A. D. (2022). Aduhelm,
a novel anti-amyloid monoclonal antibody, for the treatment of Alzheimer’s
Disease: A comprehensive review. Health Psychol.
Res..

[5] Mitra A., Sarkar N. (2020). Sequence and structure-based
peptides as potent amyloid
inhibitors: A review. Arch. Biochem. Biophys..

[6] Ren B., Liu Y., Zhang Y., Cai Y., Gong X., Chang Y., Xu L., Zheng J. (2018). Genistein:
A Dual Inhibitor of Both Amyloid beta and
Human Islet Amylin Peptides. ACS Chem. Neurosci..

[7] Gomes L. M., Bataglioli J. C., Storr T. (2020). Metal complexes that bind to the
amyloid-β peptide of relevance to Alzheimer’s disease. Coord. Chem. Rev..

[8] Lin H.-C., Ho M.-Y., Tsen C.-M., Huang C.-C., Wu C.-C., Huang Y.-J., Hsiao I.-L., Chuang C.-Y. (2017). From the cover:
comparative proteomics reveals silver nanoparticles alter fatty acid
metabolism and amyloid beta clearance for neuronal apoptosis in a
triple cell coculture model of the blood–brain barrier. Toxicol. Sci..

[9] Giorgetti S., Greco C., Tortora P., Aprile F. A. (2018). Targeting Amyloid
Aggregation: An Overview of Strategies and Mechanisms. Int. J. Mol. Sci..

[10] Aware C. B., Patil D. N., Suryawanshi S. S., Mali P. R., Rane M. R., Gurav R. G., Jadhav J. P. (2022). Natural
bioactive products as promising
therapeutics: A review of natural product-based drug development. S. Afr. J. Bot..

[11] Chiorcea-Paquim A. M., Enache T. A., De Souza Gil E., Oliveira-Brett A. M. (2020). Natural
phenolic antioxidants electrochemistry: Towards a new food science
methodology. Compr. Rev. Food Sci. Food Saf..

[12] Ren Z., Sun S., Sun R., Cui G., Hong L., Rao B., Li A., Yu Z., Kan Q., Mao Z. (2020). A Metal–Polyphenol-Coordinated
Nanomedicine for Synergistic Cascade Cancer Chemotherapy and Chemodynamic
Therapy. Adv. Mater..

[13] Tian Y., Li C., Xue W., Huang L., Wang Z. (2023). Natural immunomodulating
substances used for alleviating food allergy. Crit. Rev. Food Sci. Nutr..

[14] Andrés C. M. C., Pérez de la Lastra J. M., Juan C. A., Plou F. J., Pérez-Lebeña E. (2023). Polyphenols
as antioxidant/pro-oxidant
compounds and donors of reducing species: Relationship with human
antioxidant metabolism. Processes.

[15] Abu-Farich B., Hamarshi H., Masalha M., Kmail A., Aboulghazi A., El Ouassete M., Imtara H., Lyoussi B., Saad B. (2024). Polyphenol
contents, antibacterial and antioxidant effects of four palestinian
honey samples, and their anticancer effects on human breast cancer
cells. J. Pure Appl. Microbiol..

[16] Arias-Sánchez R. A., Torner L., Fenton Navarro B. (2023). Polyphenols
and neurodegenerative
diseases: potential effects and mechanisms of neuroprotection. Molecules.

[17] Velásquez-Jiménez D., Corella-Salazar D. A., Zuñiga-Martínez B. S., Domínguez-Avila J. A., Montiel-Herrera M., Salazar-López N. J., Rodrigo-Garcia J., Villegas-Ochoa M. A., González-Aguilar G. A. (2021). Phenolic compounds
that cross the blood–brain barrier exert positive health effects
as central nervous system antioxidants. Food
Funct..

[18] Shaham-Niv S., Rehak P., Zaguri D., Levin A., Adler-Abramovich L., Vuković L., Král P., Gazit E. (2018). Differential inhibition
of metabolite amyloid formation by generic fibrillation-modifying
polyphenols. Commun. Chem..

[19] Freyssin A., Page G., Fauconneau B., Rioux Bilan A. (2018). Natural polyphenols
effects on protein aggregates in Alzheimer’s and Parkinson’s
prion-like diseases. Neural Regen Res..

[20] Salisbury D., Bronas U. (2015). Reactive oxygen and nitrogen species: impact on endothelial
dysfunction. Nurs. Res..

[21] Ahmad, A. ; Ahmad, V. ; Zamzami, M. A. ; Chaudhary, H. ; Baothman, O. A. ; Hosawi, S. ; Kashif, M. ; Akhtar, M. S. ; Khan, M. J. Introduction and classification of natural polyphenols Polyphenols-Based Nanotherapeutics For Cancer Management Springer 2021 1–16 10.1007/978-981-16-4935-6_1

[22] Heydarzadeh S., Kia S. K., Zarkesh M., Pakizehkar S., Hosseinzadeh S., Hedayati M. (2022). The Cross-Talk between
Polyphenols
and the Target Enzymes Related to Oxidative Stress-Induced Thyroid
Cancer. Oxid. Med. Cell. Longev..

[23] Yan Z., Zhong Y., Duan Y., Chen Q., Li F. (2020). Antioxidant
mechanism of tea polyphenols and its impact on health benefits. Anim. Nutr..

[24] Rudrapal M., Khairnar S. J., Khan J., Dukhyil A. B., Ansari M. A., Alomary M. N., Alshabrmi F. M., Palai S., Deb P. K., Devi R. (2022). Dietary polyphenols
and their role in oxidative stress-induced human
diseases: Insights into protective effects, antioxidant potentials
and mechanism (s) of action. Front. Pharmacol..

[25] Goncalves P. B., Sodero A. C. R., Cordeiro Y. (2024). Natural products
targeting amyloid-beta
oligomer neurotoxicity in Alzheimer’s disease. Eur. J. Med. Chem..

[26] Fernandes L., Cardim-Pires T. R., Foguel D., Palhano F. L. (2021). Green Tea Polyphenol
Epigallocatechin-Gallate in Amyloid Aggregation and Neurodegenerative
Diseases. Front. Neurosci..

[27] Serdar B., Erkmen T., Koçtürk S. (2021). Combinations
of polyphenols
disaggregate Aβ1–42 by passing through in vitro blood
brain barrier developed by endothelium, astrocyte, and differentiated
SH-SY5Y cells. Acta Neurobiol. Exp..

[28] Kobayashi H., Murata M., Kawanishi S., Oikawa S. (2020). Polyphenols with Anti-Amyloid
β Aggregation Show Potential Risk of Toxicity Via Pro-Oxidant
Properties. Int. J. Mol. Sci..

[29] Cui Z., Qu L., Zhang Q., Lu F., Liu F. (2024). Brazilin-7-2-butenoate
inhibits amyloid β-protein aggregation, alleviates cytotoxicity,
and protects Caenorhabditis elegans. Int. J.
Biol. Macromol..

[30] Kouhi Z. H., Seyedalipour B., Hosseinkhani S., Chaichi M. J. (2024). Bisdemethoxycurcumin,
a novel potent polyphenolic compound, effectively inhibits the formation
of amyloid aggregates in ALS-associated hSOD1 mutant (L38R). Int. J. Biol. Macromol..

[31] Gea-Gonzalez A., Hernandez-Garcia S., Henarejos-Escudero P., Martinez-Rodriguez P., Garcia-Carmona F., Gandia-Herrero F. (2022). Polyphenols from traditional Chinese
medicine and Mediterranean diet are effective against Abeta toxicity
in vitro and in vivo in Caenorhabditis elegans. Food Funct..

[32] Yu L., Li X., Shi T., Li N., Zhang D., Liu X., Xiao Y., Liu X., Petersen R. B., Xue W., Yu Y. V., Hu D. S., Xu L., Chen H., Zheng L., Huang K., Peng A. (2025). Identification of novel
phenolic inhibitors from traditional Chinese medicine against toxic
alpha-synuclein aggregation via regulating phase separation. Int. J. Biol. Macromol..

[33] López-Cárdenas, F. G. ; Pérez-Jiménez, J. ; Mateos-Briz, R. ; Zamora-Gasga, V. M. ; Sánchez-Burgos, J. A. ; Sáyago-Ayerdi, S. G. Advances in mangiferin: Biosynthetic pathways, bioavailability and bioactivity. In Handbook of Dietary Flavonoids; Springer, 2023; pp. 1–37.

[34] Benard O., Chi Y. (2015). Medicinal properties of mangiferin, structural features, derivative
synthesis, pharmacokinetics and biological activities. Mini-Rev. Med. Chem..

[35] Das J., Ghosh J., Roy A., Sil P. C. (2012). Mangiferin exerts
hepatoprotective activity against D-galactosamine induced acute toxicity
and oxidative/nitrosative stress via Nrf2–NFκB pathways. Toxicol. Appl. Pharmacol..

[36] Muruganandan S., Lal J., Gupta P. (2005). Immunotherapeutic
effects of mangiferin mediated by
the inhibition of oxidative stress to activated lymphocytes, neutrophils
and macrophages. Toxicology.

[37] Alberdi, E. ; Ruiz, A. ; Sánchez-Gómez, M. V. ; Capetillo-Zarate, E. ; Matute, C. Polyphenols attenuate mitochondrial dysfunction induced by amyloid peptides. Mitochondrial Physiology And Vegetal Molecules, Academic Press, 2021, 317, 337, 10.1016/B978-0-12-821562-3.00003-4

[38] Mei S., Ma H., Chen X. (2021). Anticancer and anti-inflammatory
properties of mangiferin:
A review of its molecular mechanisms. Food Chem.
Toxicol..

[39] Rahmani A. H., Almatroudi A., Allemailem K. S., Alharbi H. O. A., Alwanian W. M., Alhunayhani B. A., Algahtani M., Theyab A., Almansour N. M., Algefary A. N. (2023). Role of Mangiferin in Management of Cancers
through Modulation of Signal Transduction Pathways. Biomedicines.

[40] Xi J. S., Wang Y. F., Long X. X., Ma Y. (2018). Mangiferin Potentiates
Neuroprotection by Isoflurane in Neonatal Hypoxic Brain Injury by
Reducing Oxidative Stress and Activation of Phosphatidylinositol-3-Kinase/Akt/Mammalian
Target of Rapamycin (PI3K/Akt/mTOR) Signaling. Med. Sci. Monit..

[41] Saviano A., Raucci F., Casillo G. M., Mansour A. A., Piccolo V., Montesano C., Smimmo M., Vellecco V., Capasso G., Boscaino A., Summa V., Mascolo N., Iqbal A. J., Sorrentino R., d’Emmanuele di Villa Bianca R., Bucci M., Brancaleone V., Maione F. (2022). Anti-inflammatory and
immunomodulatory activity of Mangifera indica L. reveals the modulation
of COX-2/mPGES-1 axis and Th17/Treg ratio. Pharmacol.
Res..

[42] Xi J.-S., Wang Y.-F., Long X.-X., Ma Y. (2018). Mangiferin potentiates
neuroprotection by isoflurane in neonatal hypoxic brain injury by
reducing oxidative stress and activation of phosphatidylinositol-3-kinase/Akt/mammalian
target of rapamycin (PI3K/Akt/mTOR) signaling. Med. Sci. Monit..

[43] Feng S.-T., Wang Z.-Z., Yuan Y.-H., Sun H.-M., Chen N.-H., Zhang Y. (2019). Mangiferin: A multipotent
natural product preventing neurodegeneration
in Alzheimer’s and Parkinson’s disease models. Pharmacol. Res..

[44] Liu T., Song Y., Hu A. (2021). Neuroprotective mechanisms of mangiferin
in neurodegenerative diseases. Drug Dev. Res..

[45] Zhang H., Hou Y., Liu Y., Yu X., Li B., Cui H. (2010). Determination
of mangiferin in rat eyes and pharmacokinetic study in plasma after
oral administration of mangiferin-hydroxypropyl-beta-cyclodextrin
inclusion. J. Ocul. Pharmacol. Ther..

[46] Xu X. (2009). γ-Secretase
catalyzes sequential cleavages of the AβPP transmembrane domain. J. Alzheimers Dis..

[47] Sun X., Chen W.-D., Wang Y.-D. (2015). β-Amyloid: the key peptide
in the pathogenesis of Alzheimer’s disease. Front. Pharmacol..

[48] Chen X.-Q., Mobley W. C. (2019). Alzheimer disease
pathogenesis: insights from molecular
and cellular biology studies of oligomeric Aβ and tau species. Front. Neurosci..

[49] Hamley I. W. (2012). The amyloid
beta peptide: a chemist’s perspective. Role in Alzheimer’s
and fibrillization. Chem. Rev..

[50] Falini B., Mecucci C., Tiacci E., Alcalay M., Rosati R., Pasqualucci L., La Starza R., Diverio D., Colombo E., Santucci A. (2005). Cytoplasmic nucleophosmin in acute myelogenous
leukemia with a normal karyotype. N. Engl. J.
Med..

[51] Falini B., Martelli M. P., Bolli N., Sportoletti P., Liso A., Tiacci E., Haferlach T. (2011). Acute myeloid
leukemia with mutated nucleophosmin (NPM1): is it a distinct entity?. Blood.

[52] Di
Natale C., Scognamiglio P. L., Cascella R., Cecchi C., Russo A., Leone M., Penco A., Relini A., Federici L., Di Matteo A. (2015). Nucleophosmin contains
amyloidogenic regions that are able to form toxic aggregates under
physiological conditions. FASEB J..

[53] Scognamiglio P. L., Di Natale C., Leone M., Cascella R., Cecchi C., Lirussi L., Antoniali G., Riccardi D., Morelli G., Tell G. (2016). Destabilisation, aggregation, toxicity and cytosolic
mislocalisation of nucleophosmin regions associated with acute myeloid
leukemia. Oncotarget.

[54] Russo A., Diaferia C., La Manna S., Giannini C., Sibillano T., Accardo A., Morelli G., Novellino E., Marasco D. (2017). Insights into amyloid-like aggregation of H2 region
of the C-terminal domain of nucleophosmin. Biochim.
Biophys. Acta.

[55] Di
Natale C., La Manna S., Malfitano A. M., Di Somma S., Florio D., Scognamiglio P. L., Novellino E., Netti P. A., Marasco D. (2019). Structural insights
into amyloid structures of the C-terminal region of nucleophosmin
1 in type A mutation of acute myeloid leukemia. Biochim. Biophys. Acta.

[56] La
Manna S., Roviello V., Scognamiglio P. L., Diaferia C., Giannini C., Sibillano T., Morelli G., Novellino E., Marasco D. (2019). Amyloid fibers deriving
from the aromatic core of C-terminal domain of nucleophosmin 1. Int. J. Biol. Macromol..

[57] La
Manna S., Scognamiglio P. L., Roviello V., Borbone F., Florio D., Di Natale C., Bigi A., Cecchi C., Cascella R., Giannini C. (2019). The acute myeloid leukemia-associated
Nucleophosmin 1 gene mutations dictate amyloidogenicity of the C-terminal
domain. FEBS J..

[58] La
Manna S., Florio D., Di Natale C., Napolitano F., Malfitano A. M., Netti P. A., De Benedictis I., Marasco D. (2021). Conformational consequences of NPM1 rare mutations:
An aggregation perspective in Acute Myeloid Leukemia. Bioorg. Chem..

[59] La
Manna S., Florio D., Di Natale C., Scognamiglio P. L., Sibillano T., Netti P. A., Giannini C., Marasco D. (2021). Type F mutation of nucleophosmin 1 Acute Myeloid Leukemia:
A tale of disorder and aggregation. Int. J.
Biol. Macromol..

[60] Florio D., Roviello V., La Manna S., Napolitano F., Maria Malfitano A., Marasco D. (2022). Small molecules enhancers
of amyloid
aggregation of C-terminal domain of Nucleophosmin 1 in acute myeloid
leukemia. Bioorg. Chem..

[61] La
Manna S., Di Natale C., Panzetta V., Leone M., Mercurio F. A., Cipollone I., Monti M., Netti P. A., Ferraro G., Teran A. (2024). A Diruthenium Metallodrug
as a Potent Inhibitor of Amyloid-β Aggregation: Synergism of
Mechanisms of Action. Inorg. Chem..

[62] Di
Natale C., Florio D., Di Somma S., Di Matteo A., Federici L., Netti P. A., Morelli G., Malfitano A. M., Marasco D. (2020). Proteostasis unbalance of nucleophosmin 1 in Acute
Myeloid Leukemia: An aggregomic perspective. Int. J. Biol. Macromol..

[63] Wördehoff M. M., Hoyer W. (2018). α-Synuclein aggregation
monitored by thioflavin T fluorescence
assay. Bio-Protocol..

[64] Di
Natale C., La Manna S., Avitabile C., Florio D., Morelli G., Netti P. A., Marasco D. (2020). Engineered
β-hairpin scaffolds from human prion protein regions: Structural
and functional investigations of aggregates. Bioorg. Chem..

[65] Filipe V., Hawe A., Jiskoot W. (2010). Critical evaluation of Nanoparticle
Tracking Analysis (NTA) by NanoSight for the measurement of nanoparticles
and protein aggregates. Pharm. Res..

[66] Moore C., Wing R., Pham T., Jokerst J. V. (2020). Multispectral Nanoparticle
Tracking Analysis for the Real-Time and Label-Free Characterization
of Amyloid-beta Self-Assembly In Vitro. Anal.
Chem..

[67] Johansson P. K., Koelsch P. (2017). Label-free imaging of amyloids using
their intrinsic
linear and nonlinear optical properties. Biomed.
Opt. Express.

[68] Pinotsi D., Buell A. K., Dobson C. M., Schierle G. S. K., Kaminski C. F. (2013). A label-free,
quantitative assay of amyloid fibril growth based on intrinsic fluorescence. ChemBiochem.

[69] La
Manna S., Panzetta V., Di Natale C., Cipollone I., Monti M., Netti P. A., Teran A., Sanchez-Pelaez A. E., Herrero S., Merlino A. (2024). Comparative
Analysis of the Inhibitory Mechanism of Aβ 1–42 Aggregation
by Diruthenium Complexes. Inorg. Chem..

[70] Saviano A., Schettino A., Iaccarino N., Mansour A. A., Begum J., Marigliano N., Raucci F., Romano F., Riccardi G., Mitidieri E., d’Emmanuele di Villa Bianca R., Bello I., Panza E., Smimmo M., Vellecco V., Rimmer P., Cheesbrough J., Zhi Z., Iqbal T. H., Pieretti S., D’Amore V. M., Marinelli L., La Pietra V., Sorrentino R., Costa L., Caso F., Scarpa R., Cirino G., Randazzo A., Bucci M., McGettrick H. M., Iqbal A. J., Maione F. (2024). A reverse translational
approach reveals the protective roles of Mangifera indica in inflammatory
bowel disease. J. Autoimmun..

[71] Bachurski D., Schuldner M., Nguyen P. H., Malz A., Reiners K. S., Grenzi P. C., Babatz F., Schauss A. C., Hansen H. P., Hallek M. (2019). Extracellular vesicle
measurements with nanoparticle
tracking analysis – An accuracy and repeatability comparison
between NanoSight NS300 and ZetaView. J. Extracell.
Vesicles.

[72] Chan H. H., Leong C. O., Lim C. L., Koh R. Y. (2022). Roles of receptor-interacting
protein kinase 1 in SH-SY5Y cells with beta amyloid-induced neurotoxicity. J. Cell. Mol. Med..

[73] Florio D., Annunziata A., Panzetta V., Netti P. A., Ruffo F., Marasco D. (2024). eta­(6)-Arene
Ru­(II) Complexes as Modulators of Amyloid
Aggregation. Inorg. Chem..

[74] Saviano A., Apta B., Tull S., Pezhman L., Fatima A., Sevim M., Mete A., Chimen M., Schettino A., Marigliano N., McGettrick H. M., Iqbal A. J., Maione F., Rainger G. E. (2025). PEPITEM,
its tripeptide pharmacophores and their peptidomimetic
analogues regulate the inflammatory response through parenteral and
topical dosing in models of peritonitis and psoriasis. Pharmacol. Res..

[75] Fan Q., Liu Y., Wang X., Zhang Z., Fu Y., Liu L., Wang P., Ma H., Ma H., Seeram N. P., Zheng J., Zhou F. (2020). Ginnalin A Inhibits Aggregation,
Reverses Fibrillogenesis, and Alleviates Cytotoxicity of Amyloid beta(1–42). ACS Chem. Neurosci..

[76] Pagano K., Tomaselli S., Molinari H., Ragona L. (2020). Natural Compounds as
Inhibitors of Abeta Peptide Aggregation: Chemical Requirements and
Molecular Mechanisms. Front. Neurosci..

