# Supplementary material for: Mangiferin as a Novel In Vitro Polyphenolic Inhibitor of Amyloid Aggregation
Source: ACS Omega. 2025 Oct 28;10(44):52773–82. doi: 10.1021/acsomega.5c06703 (PMC12612962; doi:10.1021/acsomega.5c06703)
Supplement: Supplementary file 1 [file ao5c06703_si_001.pdf]

SUPPORTING INFORMATION

Mangiferin as a novel *in vitro* polyphenolic inhibitor of amyloid aggregation

Daniele Florio[1], Enrico Gallo[1], Anella Saviano[2], Anna Schettino[2], Noemi Marigliano[2], Ilaria Leone[1], Francesco Maione[2, 3] and Daniela Marasco[4*]*

[1]IRCSS SYNLAB SDN, via G. Ferraris 144, 80146, Naples, Italy.

[2]ImmunoPharmaLab, Department of Pharmacy, School of Medicine and Surgery, University of Naples Federico II, Via Domenico Montesano 49, 80131, Naples, Italy.

[3]Nutraceuticals and Functional Foods Task Force, University of Naples Federico II, Via Domenico Montesano 49, 80131, Naples, Italy.

[4]Department of Pharmacy, School of Medicine and Surgery, University of Naples Federico II, Via Domenico Montesano 49, 80131, Naples, Italy.

[†]Co-corresponding authors:

Email: daniele.florio@synlab.it; daniela.marasco@unina.it..

List of Supplementary Material

Figure S1. SEM micrograph after 48 h of aggregation of the **MGF** compound. Overviews of the surface of samples at (**A**) 500 µm and (**B**) 50 µm.

Figure S2. Fluorescence microscopy images (λ_{exc}=359 nm, λ_{em}=461nm): Aβ_{1-42} in absence (**A**) and in the presence of **MGF** (**A'**) after 24 h of aggregation; Cterm_mutA in absence (**B**) and in the presence of **MGF** (**B'**) after 48 h of aggregation. Samples were dropped on glass slides and air-dried at room temperature. Surface overviews at 10 µm.

Figure S3. Bright field images of: Aβ_{1-42} in absence (**A**) and in the presence of **MGF** (**A'**); Cterm_mutA in absence (**B**) and in the presence of **MGF** (**B'**); **MGF** alone (**C**). Samples were drop-cast on glass slides and air-dried at room temperature. The scale bar of the images corresponds to 10 µm.

Figure S4. *In vitro* cytotoxicity, evaluated by MTT assay, for **MGF** was performed on SH-SY5Y human neuroblastoma cell line. Cells were treated with **MGF** at selected concentrations (50, 80, 250 and 400 µM) for 24 h after stirring at two different times 0, 24. The dotted lines indicate the threshold for 75% cell viability. Data are expressed as cell viability (% of control) and presented as means ± S.D. of 3 independent experiments.

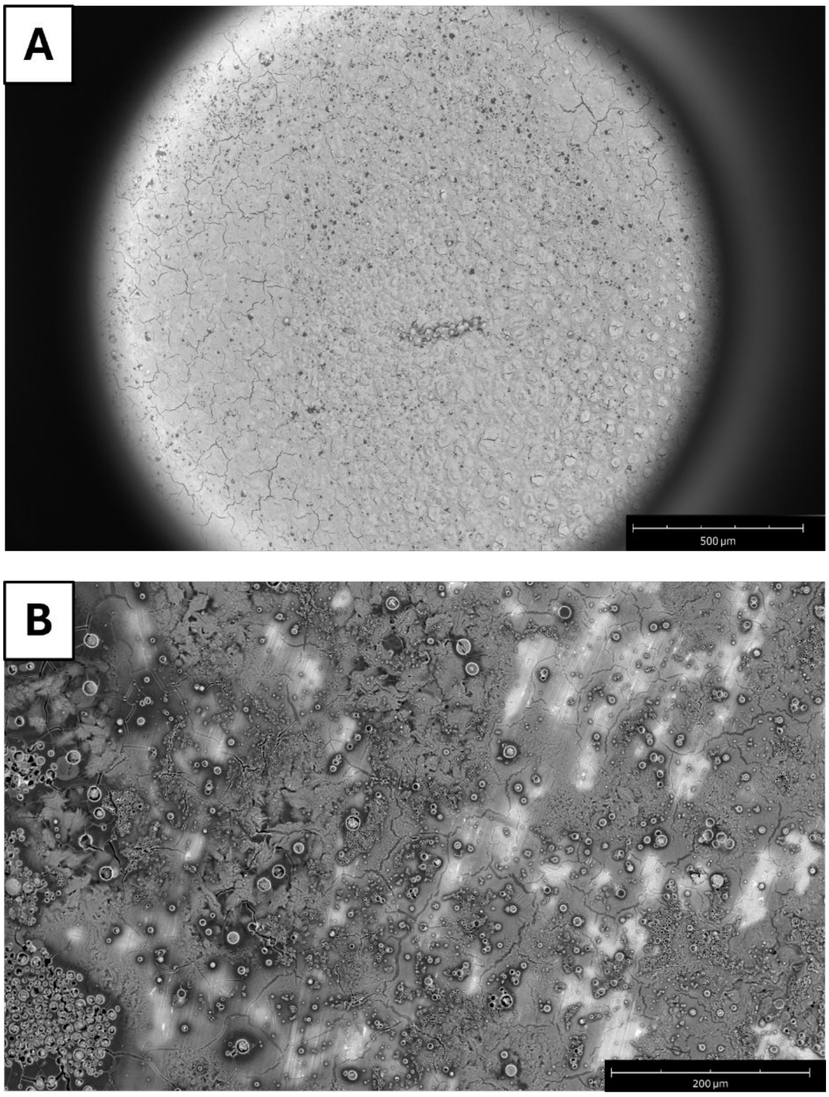

Figure S1. SEM micrograph after 48 h of aggregation of the **MGF** compound. Overviews of the surface of samples at (**A**) 500 µm and (**B**) 50 µm.

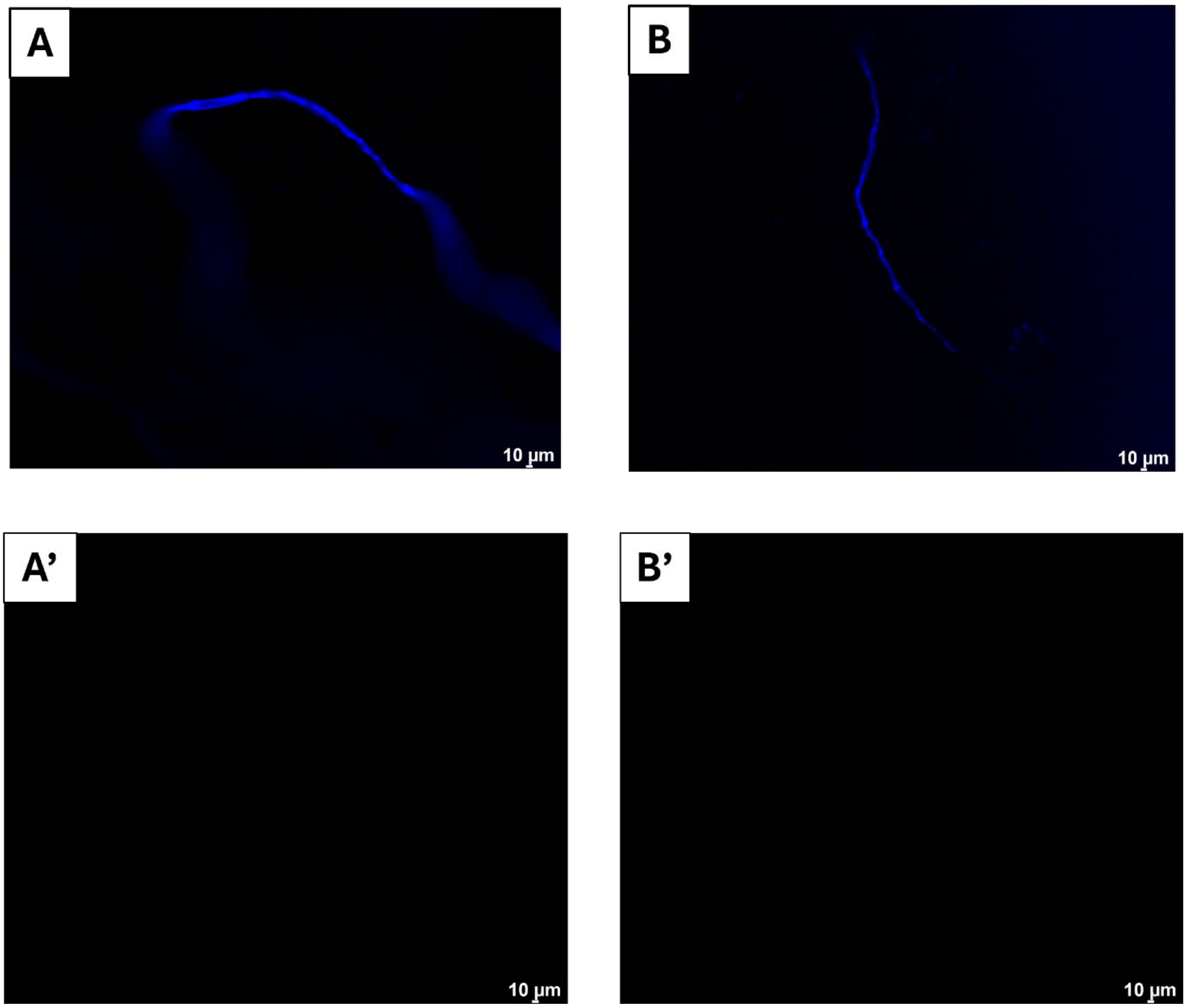

Figure S2. Fluorescence microscopy images (λ_{exc}=359 nm, λ_{em}=461nm): Aβ_{1-42} in absence (**A**) and in the presence of **MGF** (**A'**) after 24 h of aggregation; Cterm_mutA in absence (**B**) and in the presence of **MGF** (**B'**) after 48 h of aggregation. Samples were dropped on glass slides and air-dried at room temperature. Surface overviews at 10 µm.

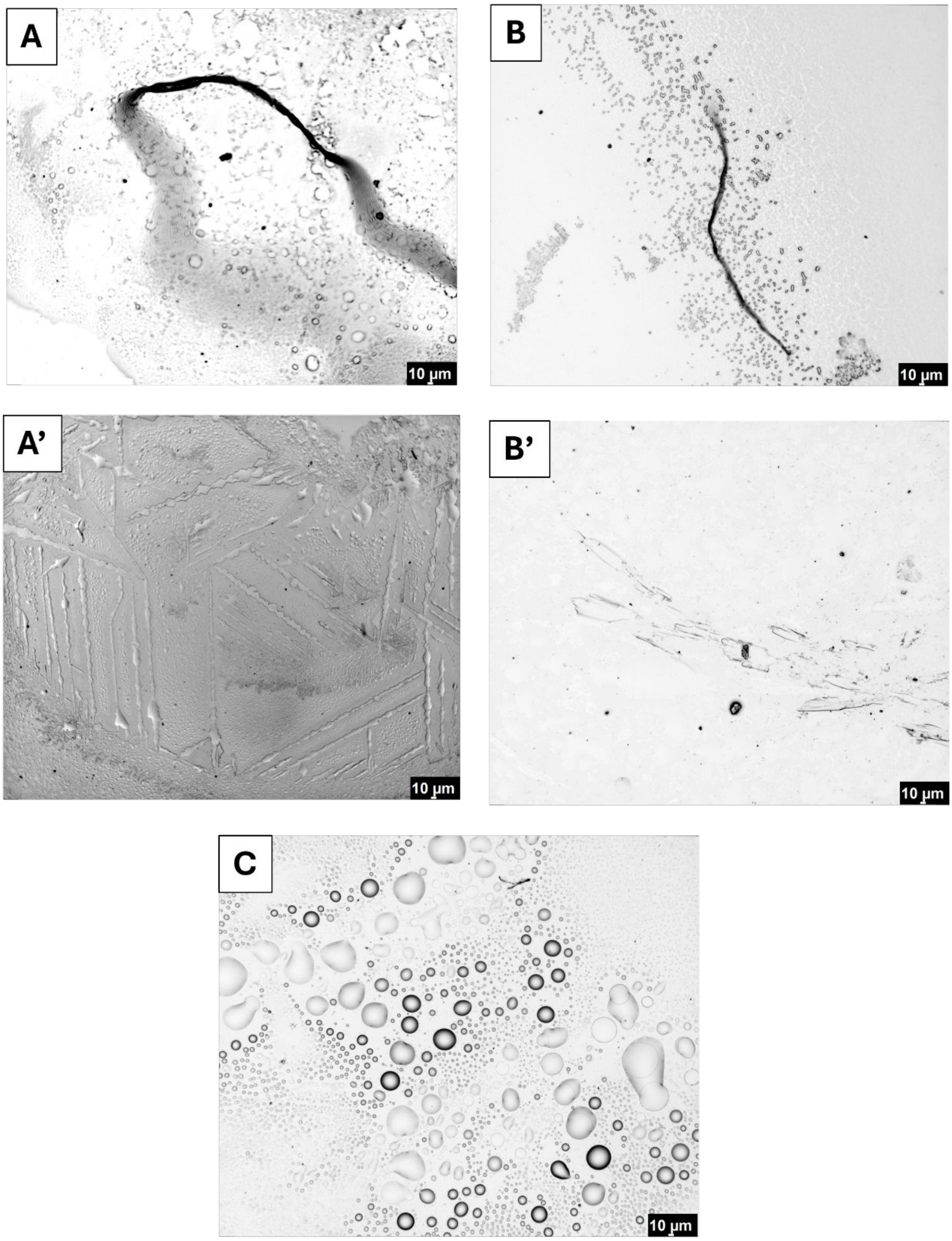

Figure S3. Bright field images of: A$\beta_{1\text{-}42}$ in absence (**A**) and in the presence of **MGF** (**A'**); Cterm_mutA in absence (**B**) and in the presence of **MGF** (**B'**); **MGF** alone (**C**). Samples were drop-cast on glass slides and air-dried at room temperature. The scale bar of the images corresponds to 10 µm.

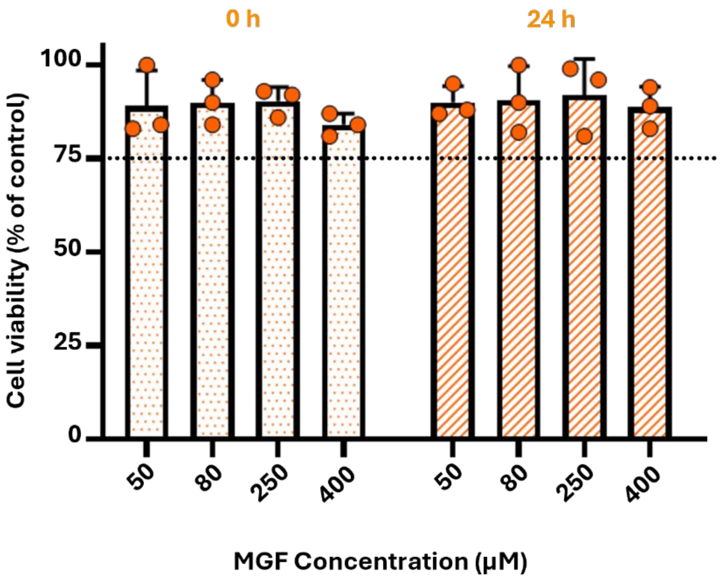

Figure S4. *In vitro* cytotoxicity, evaluated by MTT assay, for **MGF** was performed on SH-SY5Y human neuroblastoma cell line. Cells were treated with **MGF** at selected concentrations (50, 80, 250 and 400 μM) for 24 h after stirring at two different times 0, 24. The dotted lines indicate the threshold for 75% cell viability. Data are expressed as cell viability (% of control) and presented as means ± S.D. of 3 independent experiments.
